# Multiplex Lateral Flow Assay and the Sample Preparation Method for the Simultaneous Detection of Three Marine Toxins

**DOI:** 10.1021/acs.est.2c02339

**Published:** 2022-08-11

**Authors:** Clare Mills, Michael J. Dillon, Prabir Kumar Kulabhusan, Diana Senovilla-Herrero, Katrina Campbell

**Affiliations:** † Institute for Global Food Security, School of Biological Sciences, Queen’s University Belfast, 19 Chlorine Gardens, Belfast BT9 5DL, U.K.; ‡ Faculty of Health, Peninsula Medical School, University of Plymouth, Plymouth PL4 8AA, U.K.; § Kavli Institute for NanoScience Discovery, Department of Physics, New Biochemistry Building, University of Oxford, Dorothy Hodgkin Rd, Oxford OX13QU, U.K.

**Keywords:** marine toxins, lateral flow, shellfish, saxitoxin, domoic acid, okadaic acid

## Abstract

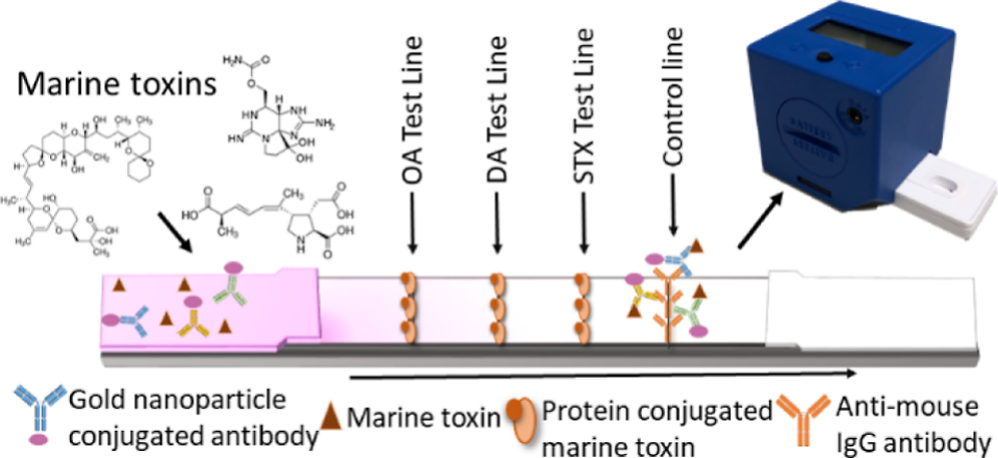

A multiplex lateral flow immunoassay (LFA) has been developed to detect the primary marine biotoxin groups: amnesic shellfish poisoning toxins, paralytic shellfish poisoning toxins, and diarrhetic shellfish poisoning toxins. The performance characteristics of the multiplex LFA were evaluated for its suitability as a screening method for the detection of toxins in shellfish. The marine toxin-specific antibodies were class-specific, and there was no cross-reactivity between the three toxin groups. The test is capable of detecting all three marine toxin groups, with working ranges of 0.2–1.5, 2.5–65.0, and 8.2–140.3 ng/mL for okadaic acid, saxitoxin, and domoic acid, respectively. This allows the multiplex LFA to detect all three toxin groups at the EU regulatory limits, with a single sample extraction method and dilution volume. No matrix effects were observed on the performance of the LFA with mussel samples spiked with toxins. The developed LFA uses a simple and pocket-sized, portable Cube Reader to provide an accurate result. We also evaluated the use of this Cube Reader with commercially available monoplex lateral flow assays for marine toxins.

## Abstract 2

Field-suitable and multiplex detection methods for marine toxins are urgently needed. The first lateral flow immunoassay to detect three marine toxins has been developed and could make important contributions to shellfish safety.

## Introduction

**Figure 1:**
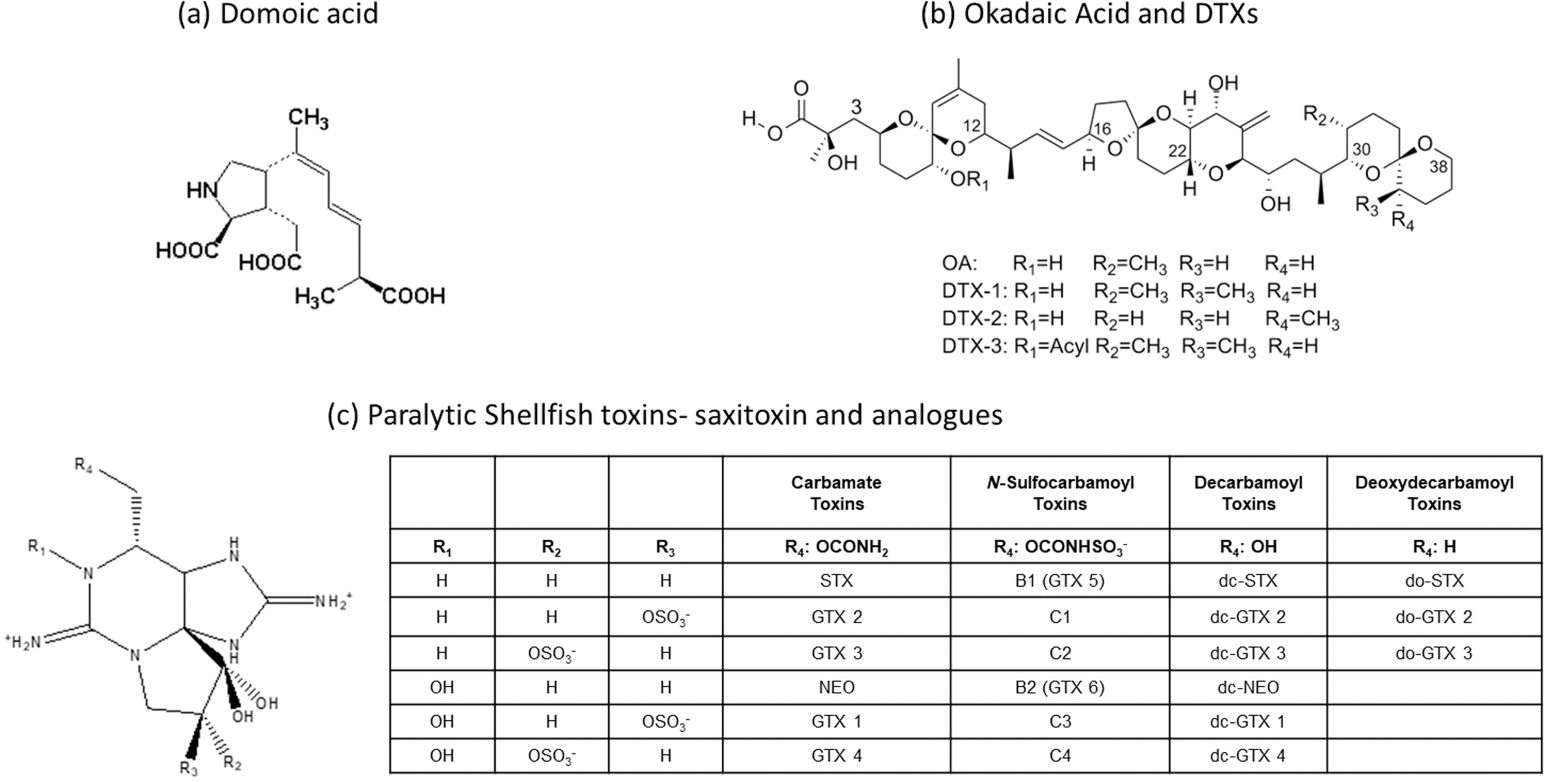
Chemical structure of the three main groups of marine toxins. (A) ASP toxin, DA. (B) DSP toxins, OA and dinophysistoxins. (C) PSP toxins, saxitoxin and its derivatives.

Marine toxins are natural compounds, produced by microalgal species during harmful algal blooms (HABs) that accumulate in shellfish. They are a threat to human health and frequently hinder the production of shellfish, causing significant economic losses.^1^ For example, the United States seafood industry reported a predicted 900 million USD annual loss due to HABs in 2016. This was a result of delays in shellfish harvesting, sales, seeding of new stock, and the destruction of contaminated stock.^2^ Similarly, in Ireland, a review of the rope mussel industry found that marine toxins were the number one factor influencing profitability.^3^ The three main groups of marine toxins are named by their symptoms; amnesic shellfish poisoning (ASP), diarrhetic shellfish poisoning (DSP), and paralytic shellfish poisoning (PSP). PSP is associated with several saxitoxin derivatives (STX), DSPs include okadaic acid (OA) and dinophysistoxins (DTXs), and ASP is caused by domoic acid (DA)^1^ (Figure 1). Many areas worldwide have enacted regulations on the maximum permitted levels of marine toxins in seafood. In the European Union, seafood must not contain marine toxins exceeding the following limits: 800 μg/kg STX equivalents, 20 mg/kg DA, or 160 μg/kg OA equivalents.^4^

Marine toxin detection has advanced significantly from the use of the mouse bioassay to analytical techniques, such as high-performance liquid chromatography with tandem mass spectrometry.^5^ However, these techniques are costly, requiring expensive laboratory equipment, specialized staff, and lengthy procedures. There is an ever-increasing use of on-site screening methods to rapidly confirm the absence of toxins in produce or indicate the need for further analysis. Sensitive, multiplex, accurate, real-time, rapid, and point-of-site testing methods suitable for novice users and low-tech environments which can be validated and used internationally is an urgent objective within the industry.^2,6^ Rapid on-site screening methods must also be inexpensive with minimal set up costs for the small- and medium-sized enterprises that dominate the shellfish industry.

Lateral flow immunoassays (LFAs) can provide a low-cost, rapid, on-site screening method for marine toxin monitoring. LFAs have minimal requirements for equipment and are easy to use in the field by non-specialists. They are suitable for use by harvesters and processors and can be tested off-site, in remote locations. Validated and commercially available LFAs for marine toxins include Neogen Reveal 2.0 for PSP toxins in scallops, oysters, clams, mussels, and cockles,^7^ Neogen Reveal 2.0 for ASP toxin DA in mussels, scallops, oysters, clams, and cockles,^8^ and Neogen Reveal 2.0 for DSP OA group toxins in mussels, scallops, oysters, and clams.^9^ Scotia also produces a range of LFAs for marine shellfish toxin detection, including DSP,^10^ ASP,^11^ and PSP^12^ test kits. The Neogen lateral flow assays are read with an AccuScan PRO reader. Although currently, this provides a sensitive and qualitative performance to the end user, the reader requires a power source limiting its in-field use. Newer technology such as the Cube Reader (ChemBio Diagnostics, Germany) meets customer requests such as being battery-powered, providing readouts within seconds, and being handheld, pocket-sized, physically robust, and with one-button operation.^13^

Although these monoplex LFAs are available, there is a distinct lack of simple, rapid, and portable multiplex assays for marine toxins in shellfish. The co-occurrence of toxin groups, year-round production, and global trade require that sites test for multiple toxin groups. It is impractical, time-consuming, and perplexing for harvesters to screen large numbers of samples with multiple tests and different sample preparation methods.^2^ Examples of multiplex immunoassays for DA, OA, and STX detection in shellfish include an automated flow-through chemiluminescence microarray developed by Szkola et al.,^14^ a surface plasmon resonance-based assay,^15^ and a solid-phase microsphere-flow cytometry system based on Luminex xMAP technology.^16^ These multiplex assays all require large, sophisticated equipment and are unsuitable for field detection by untrained personnel. McNamee et al. used an MBio cartridge (MBio Diagnostics, Inc), which combines a planar waveguide with fluorescence to detect STX, DA, OA, and freshwater toxins, microcystin-LR and cylindrospermopsin. Although this technology uses a small easy-to-use portable reader, it has yet to be reported for detection in shellfish.^17^

Sample preparation and marine toxin extraction from seafood samples are key steps in any toxin detection assay. Differences in the solubility of marine toxins complicate the development of a single sample extraction method and multiplex assays. OA group toxins are lipophilic, while STX, its derivatives, and DA are hydrophilic. Neogen Reveal 2.0 PSP and ASP kits use a simple water extraction for detection by LFA.^7,8^ The Neogen Reveal 2.0 DSP kit uses methanol extraction with an extra alkaline hydrolysis step if the detection of DTX3 is required.^9^ In this publication, we report the first multiplex LFA for the simultaneous detection of three groups of marine toxins with a single extraction solution and dilution method. The LFA is designed to work with the Cube Reader (ChemBio Diagnostics, Germany) to give a simple yes or no answer, as to whether a sample contains amounts of STX, OA, or DA over the EU regulatory limits. The developed assay was applied for the determination of marine toxins in spiked shellfish samples. We also evaluated the use of the Cube Reader with the currently available Neogen Reveal 2.0 marine toxin LFAs, as a simpler, more field-suitable method of quantification.

## Materials and Methods

### Materials

Chemical standards of STX di-HCl, DA, and OA as certified standard reference materials were obtained from the Institute for Marine Biosciences, National Research Council, Canada. OA–OVA, STX–OVA, and DA–OVA conjugates were prepared as described in.^17^ Monoclonal antibodies (Abs) applied against STX^18−20^ and DA^21^ were previously described for cross-reactivity and assay interference. The OA monoclonal antibody was purchased from Abcam (Cambridge, UK). Goat anti-mouse IgG and gold conjugation kits (40 nm, 20 OD) were obtained from Abcam (Cambridge, UK). Tween 20, methanol, sodium acetate, and ethanol were from Sigma (Dorset, UK); phosphate buffered saline (PBS) 10× solution was purchased from Fisher Scientific (Loughborough, UK). Millipore absorbent pad Grade 222 and Millipore nitrocellulose (NC) membrane HF 120 were from Merck (Darmstadt, Germany). An adhesive backing card was obtained from DCN (CA, USA) and 280 μM filter porosity BagPage + 100 filter bags from Interscience (France). Mussel samples certified free from DA, OA, and STX were obtained from Veterinary Sciences Division of the Agri-Food and Biosciences Institute, Stormont, UK. Neogen Reveal 2.0 test kits for PSPs, DSPs, and ASPs were purchased from Neogen (Scotland).

### Apparatus

The Cube Reader used in this study was provided by ChemBio Diagnostics (Germany). A sciFLEXARRAYER S3 (Scienion, Germany) was used for printing toxin conjugates onto NC membranes. The AccuScan PRO reader, used to read the Neogen Reveal 2.0 test kits, was from Neogen (Scotland).

### Conjugation of Antibodies to Gold Nanoparticles

Monoclonal antibodies were covalently attached to 40 nm gold nanoparticles (GNPs). A gold conjugation kit was used, and the manufacturer’s instructions were followed. An additional washing step was included to remove the unconjugated antibody. Ab–GNP conjugates were diluted to OD10 in assay buffer (1× PBS, 2% Tween 20).

### Immobilization of Capture Reagents

**Figure 2:**
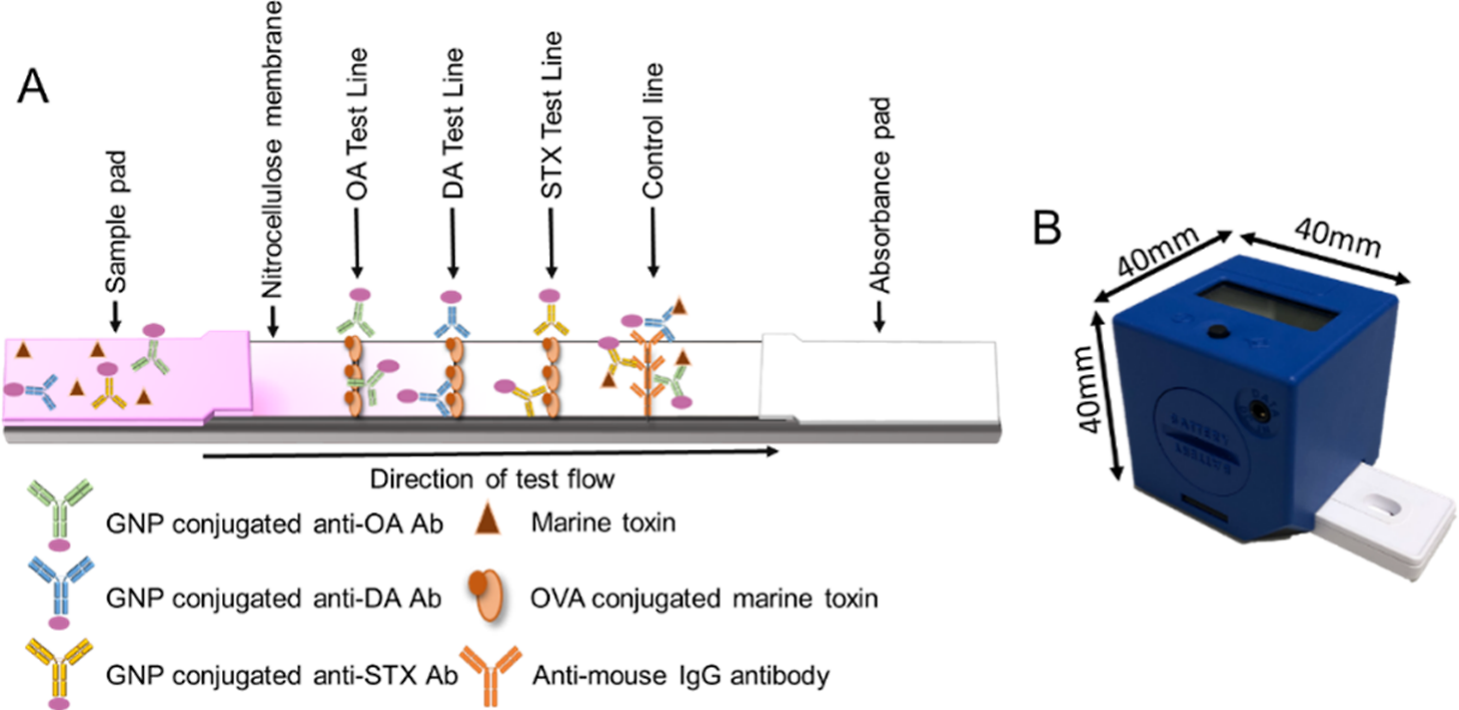
(A) Schematic of the multiplex LFA for multiple marine toxin detection. (B) Image of the ChemBio Cube Reader.

Goat anti-mouse IgG (0.5 mg/mL) in 1× PBS was applied to NC membranes as a control line, while OA–OVA, STX–OVA, and DA–OVA conjugates (0.4 mg/mL) in 1× PBS were applied as the three test lines as shown in Figure 2a. The line dispense volumes were 1 μL per cm, and lines were printed 3.5 mm apart. After dispensing, the NC membrane was dried at room temperature overnight and stored under dry conditions at room temperature until use.

### Assembly of Strips

The multiplex lateral flow strip is composed of a sample pad, NC membrane, absorbent pad, and adhesive backing card (Figure 2a). The dried NC membranes line-printed with the capture reagents were adhered to the middle section of the backing card. An absorbent pad was adhered to the end of the backing card closest to the control test line and overlapped (1 mm) with the NC membrane. The same absorbent pad was used as a sample pad, which was attached on the opposite side of the NC membrane overlapping by 1 mm. The assembled pads were cut to size (5 mm width) and mounted into a plastic housing, ready for use.

### Test Procedure

Anti-OA–GNP, anti-DA–GNP, and anti-STX–GNP were diluted 90-fold, 83-fold, and 125-fold in assay buffer, respectively. 100 μL of the antibody containing assay buffer and any indicated concentration of the toxin or mussel sample was added onto the sample pad. Test strips were left at room temperature and read using the Cube Reader after 35 min.

### Sample Preparation and Extraction

Mussel samples were homogenized and divided into 0.5 g aliquots. Samples were then spiked with fixed volumes of the relevant standard concentration of the toxin for the preparation of spiked matrix samples. The spiked 0.5 g samples were diluted in 5 mL of 0.2 M sodium acetate buffer/70% methanol or 70% ethanol/30% distilled water and left to stand for 5 min. The extract was then poured into a 280 μM micro-perforated filter bag and a metal-steam roller applied for 30 s. The filtered sample was removed from the bag and inverted in a tube 10 times. Subsequently, 10 μL of the filtrate was added to 290 μL of assay buffer. 100 μL of this was then tested, as described in the test procedure.

### Neogen Reveal 2.0 Kit Assessment

DA, OA, or STX di-HCl was spiked into Neogen Reveal 2.0 kit assay buffers for ASP, DSP, or PSP, respectively, at concentrations indicated in Figure 5. For tests read using the AccuScan PRO reader, manufacturer’s instructions were followed, and data were exported from the reader. For tests read using the Cube Reader, manufacturer’s instructions were followed until the point of the LFA reading, where LFA sticks were placed into a plastic housing suitable for the Cube Reader. The intensity of both the control and test line was then assessed with the Cube Reader.

### Data Analysis

Multiplex LFA calibration curves were constructed by plotting the ratios between the optical intensity of the toxin and blank samples, as determined by the Cube Reader, [%(*B*/*B*_0_)] against the logarithm concentrations of toxins. A four-parameter logistic regression model was plotted using GraphPad Prism 6 (GraphPad Software, USA). Displayed error bars represent the standard deviation (SD) of the mean signals (*n*) obtained as indicated. The limit of detection (LOD) (IC_10_), working range (IC_20_–IC_80_), and midpoint (IC_50_) were interpolated from the four-parameter logistic function. Percent inhibition was calculated using the equation % inhibition = 100 – *B*/*B*_0_%. For recovery calculations from spiked mussel samples, %(*B*/*B*_0_) values were used for interpolation from the calibration curves. Monoplex LFA calibration curves were constructed by plotting the ratio of the test line/control line peak area of the toxin and blank samples, as determined using the AccuScan PRO reader, %(*B*/*B*_0_) against the logarithm concentrations of toxins. All further analysis was carried out as per the multiplex LFAs.

## Results and Discussion

### Lateral Flow Assay Design and Optimization

The multiplex LFA was developed and optimized to provide quantitative detection of OA, STX, and DA within the regulatory limits for each toxin. The assay was based on a competitive reaction between the toxin conjugated to OVA and its respective antibody conjugated to GNPs. STX–OVA, OA–OVA, and DA–OVA were printed onto an NC membrane as shown in Figure 2a, as test lines. Anti-mouse IgG was printed as a control line. GNPs were chosen as probes because of their stability, ease of conjugation with antibodies, and wide use within LFAs.^7,9,22^ Assay parameters such as the toxin-OVA printing concentration and buffer, NC flow rate, absorbance/sample pad material, and run time were optimized to maintain sensitives that were suitable for each toxin’s regulatory limit. The parameter that most affected LFA performance was assay buffer composition. A high concentration of Tween 20 (2%) was required in the assay buffer to allow the anti-DA Ab-GNP to flow from the sample pad when combined with the other two Ab-GNP conjugates. The assay was designed to work with the Cube Reader (ChemBio), a small, robust, battery-operated lateral flow reader (Figure 2b).

### Assay Performance, Specificity, and Simultaneous Toxin Detection

**Figure 3:**
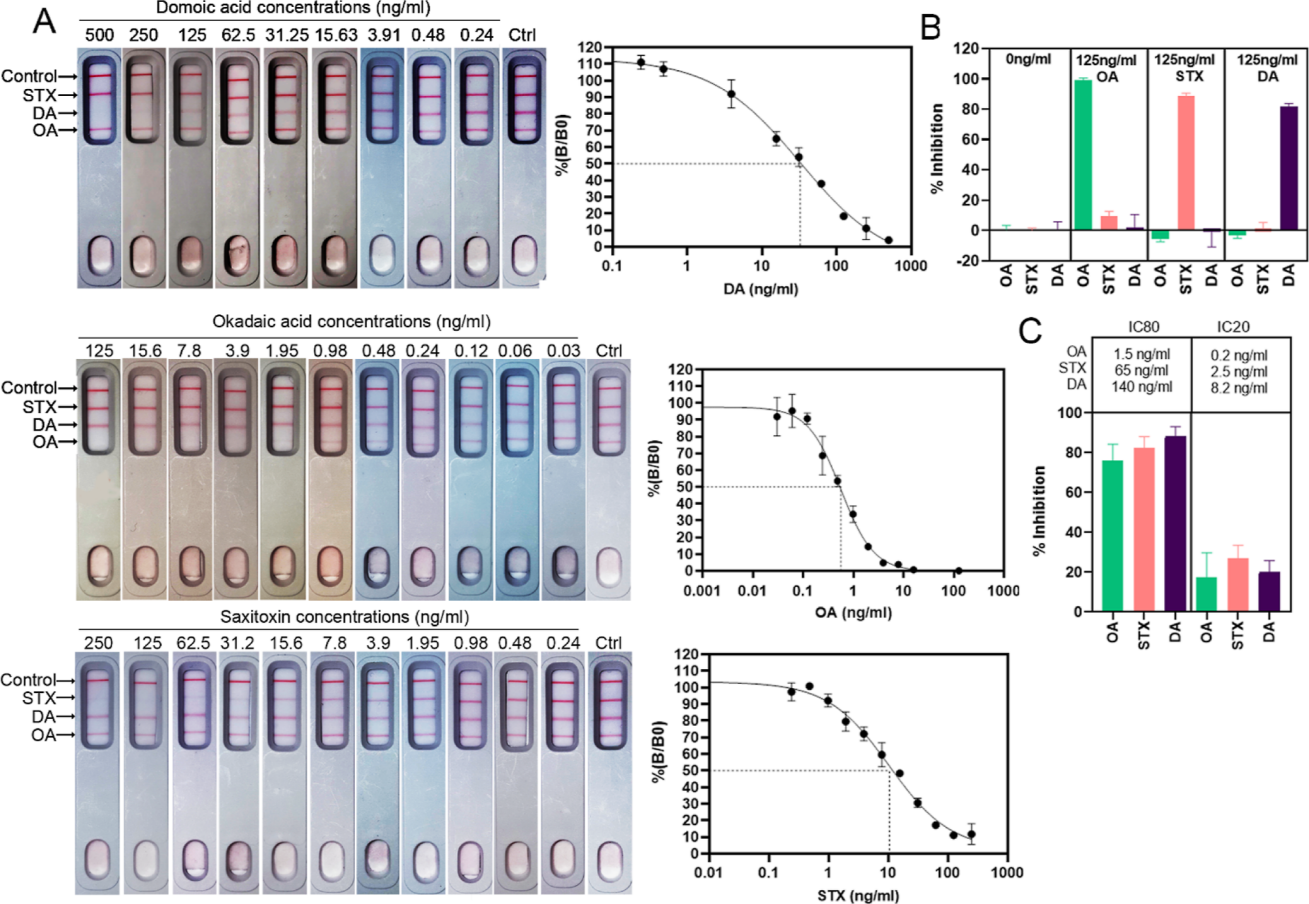
Multiplex LFA performance. (A) Individual dose–response curves for OA, STX, and DA, obtained with the developed multiplex lateral flow assay and Cube Reader. Measurements of different toxin concentrations were performed in assay buffer (*n* = 2, error bars: ±1 SD). IC_50_ is depicted as a dotted line. Corresponding images of the lateral flow devices are shown. (B) 125 ng/mL of each toxin was applied separately to the lateral flow assay in assay buffer to determine specificity. Mean normalized cube readings are shown as percent inhibition (*n* = 2, error bars: ±1 SD). (C) Mixtures of toxins in buffer at their indicated IC_80_ or IC_20_ were applied to the lateral flow assay in assay buffer to determine simultaneous detection. Mean normalized cube readings are shown as percent inhibition (*n* = 3, error bars: ±1 SD).

To assess assay performance, dose–response curves for OA, STX, and DA were generated by analyzing different concentrations of the toxins in assay buffer, using the competitive assay format and obtained optimal conditions (Figure 3a). Line intensities were quantified using the Cube Reader. For all three toxins, typical sigmoid-shaped curves with negative slopes were generated, demonstrating that test line intensity is inversely proportional to the toxin concentration. The calculated LOD for OA, STX, and DA was 0.1, 1.1, and 4.4 ng/mL, respectively. IC_50_ values were 0.6, 10.4, and 32.4 ng/mL, and working ranges were 0.2–1.5, 2.5–65.0, and 8.2–140.3 ng/mL for OA, STX, and DA, respectively. The multiplex LFA is capable of detecting the toxins within the ng/mL range, and all sensitivities are appropriate for the detection of OA, STX, and DA at the regulatory limits.

Specificity is an important parameter for the establishment of multiplex detection methods. The specificity of the designed LFA was evaluated for each toxin. Target toxins were run on the LFA at high concentrations (125 ng/mL), and their effect on each test line was assessed. Figure 3b shows that test lines specific for the corresponding toxin showed high levels of signal reduction, >80%, while no significant inhibition was seen at test lines for non-corresponding toxins. For example, the signal from the STX test line was reduced when the STX toxin was run on the LFA but not when the DA or OA toxin was run. Consequently, even at high concentrations, the marine toxins are specifically detected by the multiplex LFA. To demonstrate the ability of the developed multiplex LFA to detect OA, STX, and DA simultaneously, all three toxins were applied in mixtures at concentrations corresponding to the upper and lower limits of the working range, as calculated from single toxin detection (IC_20_ and IC_80_). All three of the different toxins were detected simultaneously using the multiplex LFA (Figure 3c). Test line intensities corresponded to those observed in LFAs performed with single toxin standard solutions, demonstrating that there is no significant cross-talk between different test lines and toxins, and multiple toxin detection does not have a negative effect on performance in comparison to single toxin detection. Taken together, these results show that all three marine toxin groups can be specifically and simultaneously distinguished by the developed LFA, regardless of the presence of other toxins.

### Detection of OA, STX, and DA in Mussel Samples

**Figure 4:**
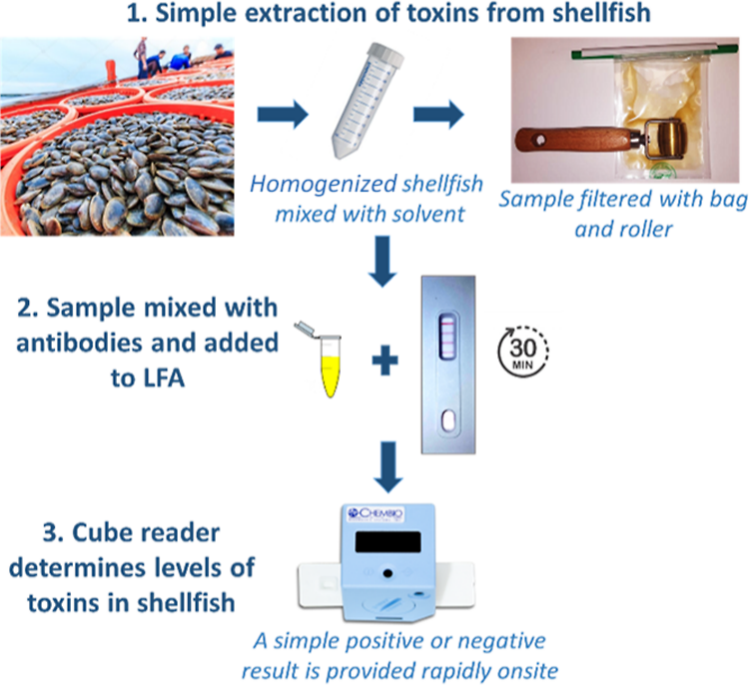
Workflow for the detection of DA, STX, and OA in shellfish samples using the multiplex lateral flow assay and cube reader. Comparison of multiplex and monoplex LFAs as quantitative methods.

To demonstrate the applicability of the multiplex LFA as a tool for monitoring toxins in seafood samples, each toxin was spiked into mussel samples and assessed. Each toxin was spiked alone at its respective regulatory limit, and all toxins were spiked together at the regulatory limits to show simultaneous detection. The different toxin working ranges meant that a single sample dilution factor (×300) could be used for all three samples, despite each toxin having different regulatory limits. The dilution factor was selected based on the region on the standard curves which would provide resolution at the regulatory level and be high enough to reduce matrix effects from the shellfish. At this dilution, the regulatory limits fell at IC_46.9_, IC_20.2_, and IC_71.1_ for OA, STX, and DA, respectively. A simple extraction method, as shown in Figure 4, was followed, using a filter bag and steamroller. Initially, an extraction buffer of 0.2 M sodium acetate, 70% methanol was tested, which has previously been demonstrated to show good recovery with all three toxins.^16^ However, this extraction buffer showed interference at the DA test line and was replaced with 70% ethanol/30% distilled water. Recovery rates of spiked mussel samples were determined from the dose–response curves obtained in assay buffer. Table 1 shows acceptable recoveries for toxins ranging from 85.8% for OA, 120.6% for DA, and 131.4% for STX. These results demonstrate that the developed multiplex LFA has the potential for use as an on-site screening method for multiple marine toxin detection in shellfish.

**Table 1:** Recovery Rates for the Detection of Various Concentrations of OA, STX, and DA Spiked in Mussel Samples with Mean %(*B*/*B*_0_) ±1SD, *n* = 3^a^

toxin	spiked conc. μg/kg	assay conc. ng/mL	determined conc. ng/mL	recovery rate (%±1 SD)
OA	160	0.5	0.5	85.8 ± 16.9
STX	800	2.7	3.5	131.4 ± 32.7
DA	20,000	66	79.6	120.6 ± 7.2

a Samples were spiked at regulatory limits and diluted at a set factor to obtain an assay concentration within the working range. Two different mussel batches were used, collected from different areas and at different times, and assays were run on at least 2 separate days for each toxin.

**Figure 5:**
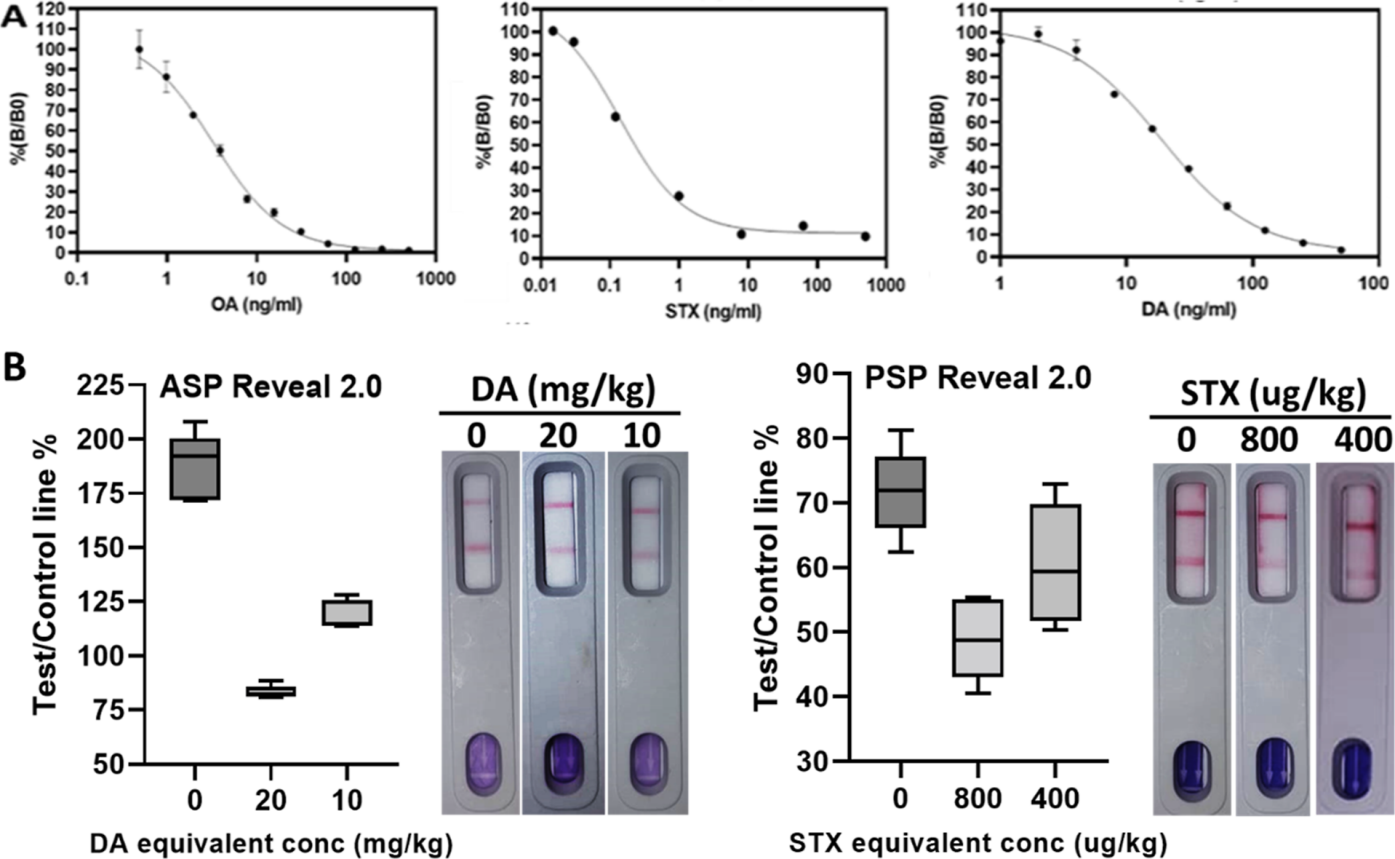
(A) Individual dose–response curves for OA, STX, and DA, obtained with the DSP, PSP, and ASP Neogen Reveal 2.0 LFAs, respectively. Measurements of different toxin concentrations were performed in assay buffer (*n* = 2, error bars: ±1 SD). (B) Quantification of Neogen Reveal 2.0 LFAs with the Cube Reader. Mean normalized cube readings are shown as the percent of test line/control line without the toxin, at toxin regulatory limits and half of the toxin regulatory limit (*n* = 5, error bars: ±1 SD).

Although manufacturers of the monoplex Neogen ASP, DSP, and PSP kits recommend their use as qualitative “yes/no” assays, the AccuScan PRO reader they are used with can provide a numerical result, which has been recommended to be made available to the end user, as a useful tool for determining toxin amounts.^11,23,24^ The occurrence of toxins remains difficult to predict owing to the complex dynamics that cause algal bloom development, their heterogeneous distribution, and rapid appearance.^2^ To provide sufficient warning to allow for changes in harvest patterns and the relocation of large quantities of shellfish, a quantitative or semi-quantitative approach would be of great use to shellfish farmers. DSP and PSP kits cannot unequivocally measure the levels of each congener of regulatory interest, due to variations in the specificity of the antibodies for different congeners, and could only be considered as semi-quantitative. In the case of ASPs, DA is the only relevant regulated toxin, and an ASP LFA could be considered fully quantitative. To compare the performance of the developed multiplex assay, as a quantitative/semi-quantitative method, with that of the monoplex Neogen kits, dose–response curves for OA, STX, and DA were generated on the Neogen kits in assay buffer using the same standards and data analysis as those performed for the multiplex kit. Line intensities were quantified using the recommended AccuScan PRO reader (Figure 5a). Table 2 summarizes parameters describing assay performance (LOD, IC_50_, and working range) obtained from the dose–response curves. Assay parameters from the developed multiplex lateral flow are included for comparison. The working range for the detection of OA in the multiplex assay was considerably narrower; however, the sensitivity was improved in comparison with the monoplex assay. For STX, the working range was wider in the multiplex LFA, but the assay was less sensitive than the monoplex LFA. The upper working range for the detection of DA in the multiplex lateral flow was nearly double with no significant loss in sensitivity in comparison with the monoplex assay. Although the loss in sensitivity for STX detection in the multiplex LFA may limit its purpose as a semi-quantitative method to provide early warning of toxin accumulation, in comparison with the monoplex LFAs, it would still be of use to provide an indication of reducing toxin levels. As OA and DA detection by the multiplex LFA has similar or improved sensitivities, they would be as useful as the monoplex LFAs in providing an indication of rising toxin levels.

**Table 2:** Assay Parameters of the Monoplex Neogen Reveal 2.0 LFAs, in Comparison with Those of the Developed Multiplex Lateral Flow Assay for the Detection of OA, STX, and DA

assay	LOD (ng/mL)	working range (ng/mL)	IC_50_ (ng/mL)
Neogen Reveal 2.0 DSP	0.8	1.3–12.7	3.8
OA Multiplex LFA	0.1	0.2–1.5	0.6
Neogen Reveal 2.0 PSP	0.04	0.1–1.7	0.2
STX Multiplex LFA	1.1	2.5–65.0	10.4
Neogen Reveal 2.0 ASP	3.6	6.6–69.3	20.5
DA Multiplex LFA	4.4	8.2–140.3	32.4

### Evaluation of the Cube Reader with Neogen LFAs

To further evaluate the utility of the Cube Reader in the detection of marine toxins, the commercially available and validated Neogen Reveal 2.0 LFAs for PSPs and ASPs were assessed with the Cube Reader, instead of the recommended AccuScan PRO reader. The Cube Reader has several advantages over the AccuScan PRO reader for in-field detection including smaller size, more physical robustness, and battery operation (Figure 2b). DA and STX di-HCl were spiked into PSP and ASP assay buffers, respectively. STX di-HCL was spiked at a concentration that is equivalent to 800 or 400 μg/kg and DA at a concentration equivalent to 20 and 10 mg/kg, that is, the EU regulatory limits or half the EU regulatory limits, respectively. Figure 5b shows that the Cube Reader was clearly able to distinguish between samples containing no toxin and the relevant toxin at the EU regulatory levels for both the PSP and ASP lateral flow assays. The Cube Reader was also clearly able to distinguish between 10 and 20 mg/kg DA on the ASP Neogen Reveal 2.0 LFAs. This illustrates that the use of the Cube Reader, with its practical advantages over the AccuScan PRO reader, could be considered for use with the ASP Neogen Reveal 2.0 LFAs. However, there was an overlap between some results when 400 and 800 μg/kg STX were tested on the PSP Neogen Reveal 2.0 LFAs. This LFA has previously been noted to produce false positives at STX equivalent concentrations close to the regulatory value.^7,23^ One study found the PSP Neogen Reveal 2.0 LFA to produce false positives, that is, report a value of >800 μg/kg in a sample containing <800 μg/kg toxic STX equivalents, in 25% of shellfish samples tested.^23^ Only one of these false positives contained <400 μg/kg STX toxic equivalents. Although the false positive results can be due to issues with differing antibody specificities between different PSP congeners and matrix effects, it is surprising that the Cube Reader cube could not accurately differentiate between these two STX concentrations and calls into question if a clearer difference in line intensities in the PSP Neogen Reveal 2.0 LFA at different STX concentrations could reduce the number of false positives.

Although LFAs for the detection of marine toxins in shellfish have proven to be an invaluable tool, there is still great room for improvement in terms of their practicality. In this report, a multiplex LFA device was developed for simultaneous detection of the three main marine toxins OA, DA, and STX. The optimized LFA sensitivities allow for the use of a single simple sample extraction method, with a single sample dilution volume to detect OA, STX, and DA at their varying EU regulatory limits. A preliminary sample extraction method has been successfully developed and tested for compatibility in the shellfish matrix. The method from sample extraction to the test result can be completed in less than 45 min. This platform offers multiple advantages over currently available monoplex LFAs or other published multiplex assays for marine toxins. Not only could it reduce the testing burden and costs for shellfish harvesters and regulatory agencies but also increase public safety. The prevalence and locations of HABs and the toxins they produce are fluctuating with our changing climate. If there is no additional workload in testing for multiple toxins rather than a single toxin, known to the area, then unexpected toxins will also be detected.

We have demonstrated the applicability of the proposed multiplex LFA as a rapid on-site, low-tech, screening tool for assessing marine toxin occurrence in shellfish. Future work must include a detailed assessment of the LFA’s stability and robustness in the field and single- and multi-laboratory validation of AOAC-accredited standards for the analysis of naturally contaminated shellfish samples and a detailed assessment of the detection of PSP and DSP congeners. Following this, the developed multiplex test could make an important contribution toward ensuring the safety of shellfish and the prevention of economic losses in many locations. In addition, we have demonstrated the utility of the Cube Reader, not only with the developed LFA but also with the Neogen Reveal 2.0 PSP and ASP lateral flow assays. The Cube Reader has several advantages over the AccuScan PRO reader for the rapid, on-site detection of marine toxins. It is battery-operated, smaller in size, and more physically robust. Future validation of these commercially available LFAs with more modern and convenient readers and their use as quantitative or semi-quantitative methods should therefore be considered, which have the potential to be of great use within the shellfish industry.

## Abbreviations

LFA: lateral flow assay
HBA: harmful algal blooms
ASP: amnesic shellfish poisoning
DSP: diarrhetic shellfish poisoning
PSP: paralytic shellfish poisoning
STX: saxitoxin
OA: okadaic acid
DTXs: dinophysistoxins
DA: domoic acid
GNPs: gold nanoparticles
LOD: limit of detection
